# miR-19b Regulates Ventricular Action Potential Duration in Zebrafish

**DOI:** 10.1038/srep36033

**Published:** 2016-11-02

**Authors:** Alexander Benz, Mandy Kossack, Dominik Auth, Claudia Seyler, Edgar Zitron, Lonny Juergensen, Hugo A. Katus, David Hassel

**Affiliations:** 1 Department of Medicine III, Cardiology, Angiology and Pneumology, University Hospital of Heidelberg, 69120 Heidelberg, Germany; 2 DZHK (German Centre for Cardiovascular Research), Partner Site Heidelberg/Mannheim, 69120 Heidelberg, Germany

## Abstract

Sudden cardiac death due to ventricular arrhythmias often caused by action potential duration (APD) prolongation is a common mode of death in heart failure (HF). microRNAs, noncoding RNAs that fine tune gene expression, are frequently dysregulated during HF, suggesting a potential involvement in the electrical remodeling process accompanying HF progression. Here, we identified miR-19b as an important regulator of heart function. Zebrafish lacking miR-19b developed severe bradycardia and reduced cardiac contractility. miR-19b deficient fish displayed increased sensitivity to AV-block, a characteristic feature of long QT syndrome in zebrafish. Patch clamp experiments from whole hearts showed that miR-19b deficient zebrafish exhibit significantly prolonged ventricular APD caused by impaired repolarization. We found that miR-19b directly and indirectly regulates the expression of crucial modulatory subunits of cardiac ion channels, and thereby modulates AP duration and shape. Interestingly, miR-19b knockdown mediated APD prolongation can rescue a genetically induced short QT phenotype. Thus, miR-19b might represent a crucial modifier of the cardiac electrical activity, and our work establishes miR-19b as a potential candidate for human long QT syndrome.

A frequent feature of cardiomyocytes from failing hearts, independent of the cause, is the prolongation of the action potential (AP) regularly caused by alterations in the functional expression of cardiac potassium and sodium channels^1^,^2^,^3^,^4^. However, the molecular mechanisms responsible for changes in ion channel expression remain elusive. Evidence exists indicating that various miRNAs, including miR-19, are differentially expressed in the dysfunctional myocardium, implicating a possible role in contributing to the progression and the characteristics of the disease^5^,^6^. While miR-19 represents an established oncogenic miRNA, knowledge on its cardiovascular function is still elusive^7^. miR-19 is transcribed as part of the miR-17-92 cluster and is encoded by two isoforms, miR-19a and miR-19b, that only differ by one nucleotide outside the seed^8^. First evidence of a potential involvement in the heart came from a study demonstrating that depletion of the miR-17~92 cluster resulted in ventricular septal defects during cardiac development^9^. Furthermore, decreased expression of specifically miR-19a and miR-19b was shown to contribute to human age-related heart failure, cardiac fibrosis, and left ventricular stiffening through direct targeting of the extracellular matrix components connective tissue growth factor (CTGF) and thrombospondin-1 (TSP-1)^10^. Ikeda *et al.* reported that miR-19a and miR-19b expression is significantly and consistently reduced in hearts from dilated cardiomyopathy (DCM) patients^5^. When overexpressed, cardiac-specific miR-17~92 transgenic animals developed cardiac hypertrophy and showed substantial susceptibility to lethal arrhythmias^11^. Two subsequent studies attributed significant contribution for both observed characteristics to miR-19a/b. Song *et al.* demonstrated that miR-19a/b positively influence cardiomyocyte hypertrophy by directly targeting MuRF-1 and atrogin-1, while a recent study showed that overexpression of miR-19a/b caused arrhythmia and bradycardia in zebrafish^12^,^13^.

Here, we used zebrafish to gain further insights into the role of miR-19 in the heart. Zebrafish proved valuable to study cardiac development and function in a human relevant context, including electrical physiology, since zebrafish AP shape and duration, heart rate, and electrocardiogram closely resemble that of humans^14^. We identified a so far unappreciated role for miR-19b in directly regulating cardiac electrical activity. Reduction of specifically miR-19b by antisense morpholino modified oligonucleotides and by CRISPR-mediated knockout was sufficient to induce severe bradycardia as well as susceptibility to arrhythmias and cardiomyopathy in zebrafish. Furthermore, miR-19b deficient zebrafish displayed increased sensitivity to action potential prolonging drugs, suggesting impaired repolarization. By patch-clamp we show that the action potentials recorded from miR-19b deficient zebrafish hearts are significantly prolonged. We further provide evidences that miR-19b modulates AP duration (APD), in part, by directly targeting KCNE4.

## Results

### miR-19 is expressed in the heart and is induced at stages of first heart activity

**Figure 1:**
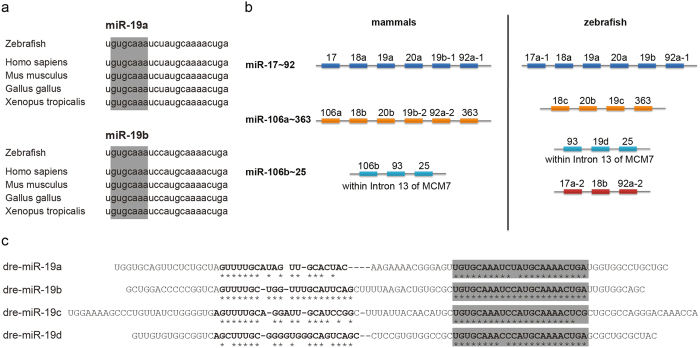
miR19 is conserved in sequence and syntheny. **(a)** The sequence of miR-19a and miR-19b is highly conserved within vertebrate species. The seed-region is shaded in grey. **(b)** miR-19-Isoforms are encoded within the miR-17-92 cluster and its paralogs the miR-106a-363- and the miR-106b-25-cluster in mammals and zebrafish. **(c)** The zebrafish encodes for four different isoforms of miR-19, miR-19a-d. The sequence of their mature isoforms differs in maximal two nucleotides. Mature sequences are shaded in grey. Conserved sequence is indicated with stars (compared to miR-19b).

**Figure 2:**
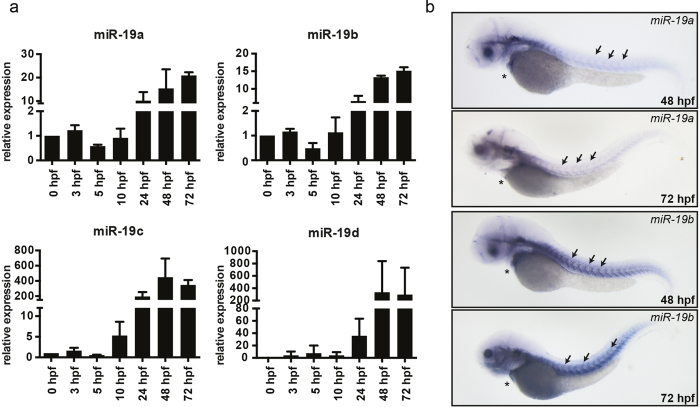
Expression of miR-19 in the zebrafish. **(a)** qRT-PCR analysis revealed that miR-19a-d expression is already detectable at very early stages of embryonic development and gets induced at 24 hpf. (±sd; n = 3 from 15 pooled embryos per sample) **(b)** *In situ* hybridization of miR-19a and miR-19b in 48 hpf and 72 hpf zebrafish embryos shows similar expression patterns in the heart (stars) and in skeletal muscle myosepts (arrows).

The sequence and the synteny of miR-19 is highly conserved among vertebrate species (Fig. 1a,b). While mammals express two isoforms, miR-19a and miR-19b, zebrafish exhibit four miR-19 isoforms encoded in four genomic loci (Fig. 1b,c). qRT-PCR analysis of single miR-19 isoforms showed detectable expression at the one-cell stage for miR-19a and miR-19b, with strong induction by 24 hpf, coinciding with the initiation of heart contractions in zebrafish (Fig. 2a). miR-19c and miR-19d are undetectable before 10 hpf. Similar to miR-19a/b, expression is induced at 24 hpf. However, despite a dramatic fold upregulation, the consistently high cT values of miR-19c/d indicate a generally low expression of these two isoforms at all developmental time points. In contrast, miR-19a/b generally present low cT values which are often similar as for the endogenous control gene, indicating relatively high expression levels. *In situ* hybridization, to detect the likely mammalian homologous miR-19a and miR-19b transcripts, based on the complete sequence homology to the mammalian isoforms and their predominant expression levels, showed similar expression patterns for both isoforms at 48 and 72 hpf with robust expression in the skeletal muscle myosepta and in the heart (Fig. 2b).

### Loss of miR-19 results in bradycardia and cardiomyopathy

**Figure 3:**
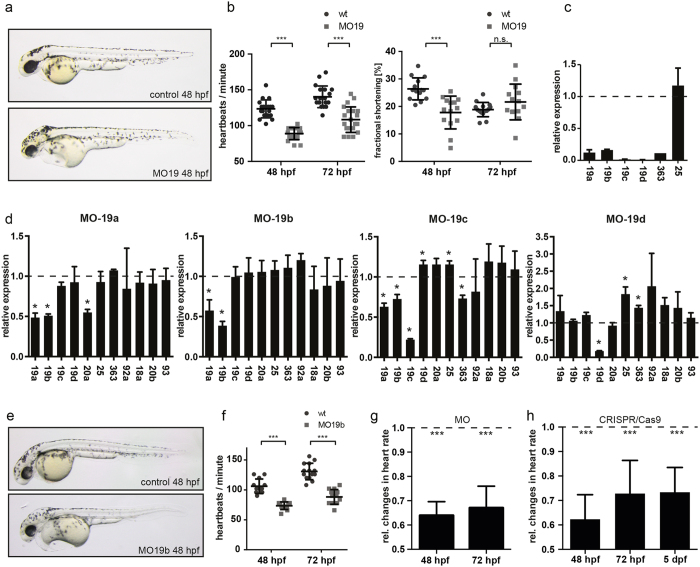
Loss of miR-19 leads to bradycardia. (**a)** Lateral brightfield images of control-injected and MO19-injected 48 hpf zebrafish embryos. Note the cardiac edema and the blood congestion at the inflow tract developed in MO19 injected embryos. **(b)** Quantification of heart rate as heart beats per minute and ventricular fractional shortening as a measure for ventricular contractility in control (wt) and MO19 injected embryos. Morpholino-mediated knockdown of miR-19 in zebrafish leads to bradycardia and impaired ventricular contractility (±sd; n ≥ 14; p < 0.005). **(c)** qRT-PCR confirmed efficient knockdown of miR-19a-d. Notice the consistent reduction in miR-363 expression (±sd; n = 45 animals from 3 independent experiments). **(d)** Specific knockdown of miR-19a, miR-19c and miR-19d altered expression of neighboring miRNAs. However, knockdown of miR-19b did not interfere with expression of neighboring miRNAs (±sd; n = 45 animals from 3 independent experiments; p < 0.05). **(e)** 48 hpf zebrafish embryos injected with control- and MO19b-morpholino revealed that miR-19b deficiency is sufficient to mimic MO19-induced phenotype with characteristics of heart dysfunction. **(f)** miR-19b deficient embryos developed bradycardia with up to 36% ± 5.7% reduced heart frequency (± = sd; n ≥ 12; p < 0.005) **(g,h)** Relative changes in heart rate normalized to controls (dotted line) indicates that CRSIPR/Cas9 mediated knockout of miR-19b caused an identical and long-term reduction in heart rate as observed for MO19b (±sd; n = 10; p < 0.005).

To investigate the effects of loss of miR-19 on heart function, we initially injected a morpholino-modified oligonucleotide targeting all four isoforms of miR-19 (MO19) into zebrafish embryos at the single-cell stage. MO19 was designed to specifically block the processing of all miR-19 isoforms into their mature and active forms (suppl. Figure 1). qRT-PCR analysis confirmed significant reduction of mature miR-19a, miR-19b, miR-19c, and miR-19d by at least 90% (Fig. 3c). miR-19 deficient zebrafish displayed signs of cardiac dysfunction with cardiac edema and blood congestion at the inflow tract (Fig. 3a). MO19 injected animals most noticeably and very consistently developed severe bradycardia with up to 28% ±6.7 reduced heart rate (Fig. 3b, suppl. Movie 1, 2). Interestingly, bradycardia was frequently, but not consistently, accompanied by significantly reduced ventricular contractility (Fig. 3b; suppl Movie 1, 2).

### Identification of miR-19b as causative isoform

Because all miR-19 isoforms are genomically closely located and polycystronically encoded on single pri-miRNA transcripts containing other miRNAs, we evaluated the impact of miR-19 knockdown on the expression of neighboring miRNAs to detect potential unintentional co-regulatory effects. We found that injection of MO19 not only profoundly reduced the expression of mature miR-19a-d, but very consistently also diminished the level of mature miR-363 (Fig. 3c). miR-363 is encoded downstream of miR-19c on one pri-miRNA transcript (Fig. 1d). To exclude that loss of miR-363 is causing or contributing to the observed miR-19 deficiency phenotype, we used a morpholino targeting specifically miR-363 processing (suppl. Figure 2A). miR-363-deficient embryos most noticeably showed severe abnormalities specifically in eye development, with dramatically reduced eye size up to complete loss of any eye structure when injected with high dosages of the MO (suppl. Figure 2C). Interestingly, MO-19 injected embryos often display noticeable defects in eye development, with frequently smaller and seemingly impaired coalescence of the ventral retina (Fig. 3a). This might be explained by the involvement of the co-regulated miR-363 in eye development in MO19 injected embryos. However, cardiac morphology and heart function, analyzed by measuring heart rate and fractional shortening, were not affected in miR-363-deficient animals (suppl. Figure 2D,E).

To ensure greater specificity in evaluating miR-19 function and to enable the identification of a single causal miR-19 isoform, we used four individual morpholinos to specifically target each miR-19 isoform exclusively (suppl. Figure 1). Therefore, the morpholinos were designed to target the pre-miR-19a-d star isoforms harboring unique nucleotide sequences for each isoform, thereby allowing specific targeting of every single miR-19 isoform individually by inhibiting the processing of the mature miRNA and the corresponding star isoform^15^,^16^. qPCR analysis revealed that reduction of either miR-19a, miR-19c, or miR-19d alone resulted in unintended altered expression of miR-20a, miR-363, or miR-25, respectively (Fig. 3d). Only injection of a MO against miR-19b specifically (MO19b) did not affect expression of any other neighboring miRNA (Fig. 3d). Importantly, MO19a or MO19b injection individually caused a reduction of mature miR-19a and miR-19b both by 50%. Given the high similarity of miR-19a and miR-19b mature sequences, this observed co-modulation detected by qPCR is likely due to the inability of the qPCR assays used to distinguish both individual isoforms. Noticeably, MO19b injection was sufficient to cause bradycardia, similar to the previously used MO with a reduction of the heart rate by 36% ± 5.7% (Fig. 2f,g). Importantly, eye morphology was unaffected in MO19b injected embryos, further confirming specificity. To further assess miR-19b causality in developing bradycardia, we deployed CRISPR/Cas9-mediated genome editing to specifically mutate miR-19b genomically and thereby impair miR-19b expression. Therefore, similar to MO19b, we decided to target a less conserved sequence within the miR-19b gene, again to not interfere with the expression of homologous miR-19-isoforms (suppl. Figure 3A). We designed a single guide RNA (sgRNA) to target the drosha-site to inhibit miR-19b procession. Noticeably, 19b-Crispants showed profoundly decreased miR-19b expression as assessed by qPCR (suppl. Figure 3B). Importantly, these animals similarly developed bradycardia with an identical reduction in heart rate by 37.7% ± 9.5% that persisted, as a consequence of the genetic deletion, even beyond 5 dpf, confirming the effect of miR-19b on heart rhythm (Fig. 3h). Consistent with previous reports, we found that overall morphology of miR-19b deficient hearts appeared to be normal, suggesting normal heart development (suppl. Figure 4)^17^.

miR-19b overexpression was previously shown to impair angiogenesis^18^,^19^,^20^. To assess whether loss of miR-19b affects angiogenesis in zebrafish during early development, we examined vessel formation in 48 and 72 hpf *Tg*(*kdrl:EGFP*)^*s843*^ transgenic zebrafish expressing green fluorescent protein (GFP) under the control of an endothelial-specific promoter, labeling all vascular endothelial cells with GFP. We found that miR-19b reduction by MO19b injection did not alter overall angiogenesis (suppl. Figure 5). These results lead us to conclude that miR-19b is involved in modulating cardiac conduction and electrical activity of the heart.

### miR-19b deficient zebrafish display prolonged action potentials

**Figure 4:**
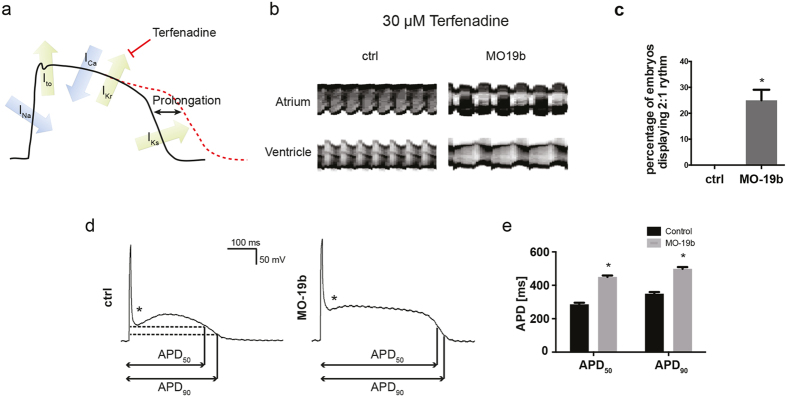
Loss of miR-19b induces prolongation of the action potential. **(a)** Schematic illustration of an action potential with major depolarizing and repolarizing currents depicted. Terfenadine inhibits I_Kr_ current, thereby prolonging the ventricular action potential. **(b)** Atrial and ventricular m-mode recorded from control and MO19b-injected *Tg*(*Myl7:GFP*) zebrafish embryos after 30 μM Terfenadine treatment. **(b,c)** 25% ± 4.1% of miR-19b deficient embryos develop an AV-Block with the ventricle skipping every other contraction, whereas control injected embryos show no phenotype. (±sd; n = 3 with ≥10 animals per group; p < 0.05) **(d,e)** Patch-clamp Experiments revealed that morpholino-mediated loss of miR-19b results in prolonged action potentials with up to 57% ± 3.8% and 39% ± 3.5% prolongation of APD_50_ and APD_90_, respectively (±sd; n ≥ 10 from 5 hearts; p < 0.05). Additionally, the notch appeared to be less pronounced (stars).

**Figure 5:**
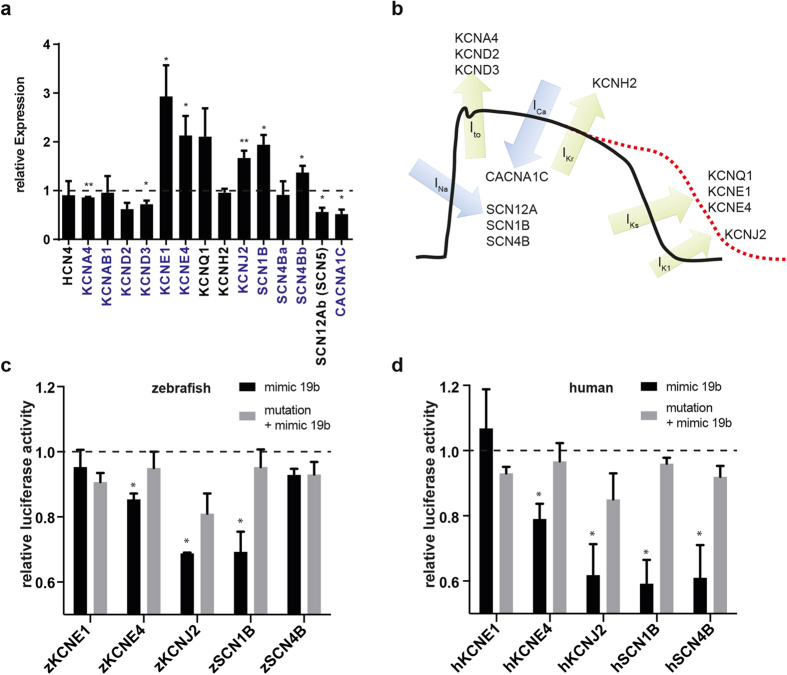
Loss of miR-19b results in dysregulation of cardiac ion currents. **(a)** qRT-PCR revealed dysregulation of various cardiac ion channels in miR-19b-deficient zebrafish. Transcripts with a predicted miR-19b binding site in its 3′-UTR are indicated in blue (±sd; n = 3 from 15 pooled embryos per sample; p < 0,05). **(b)** Illustration of a mammalian action potential with corresponding currents and genes. **(c,d)** Potential miR-19b targets were tested for direct regulation by a dual luciferase reporter gene assay. 3′UTR of indicated zebrafish **(c)** and human **(d)** genes was cloned downstream of a luciferase gene. Luciferase expression was normalized to renilla expression. As control, target sites were mutated to reverse miR-19b-induced repression. KCNE4, KCNJ2 and SCN1B proved to be direct targets in zebrafish and in humans. Additionally, the 3′UTR of human SCN4B showed responsiveness to miR-19b (±sd; n = 3; p < 0.05).

The reduced heart rate might either be caused by a dysfunction of the sinus node or by prolongation of the APD. Since prolongation of the APD is common in human cardiomyopathy, and pharmacologically and genetically induced prolongation of the APD cause bradycardia in zebrafish, we decided to initially test for this^1^,^2^,^3^,^4^,^21^. To assess whether miR-19b knockdown hearts develop prolonged APs, we performed a sensitizing experiment using Terfenadine to pharmacologically block the I_Kr_ current, thereby artificially prolonging the AP (Fig. 4a). AP prolongation beyond a specific threshold in zebrafish produces a very characteristic and easy-to-observe atrio-ventricular block phenotype, with the ventricle only contracting after every other atrial contraction, as described for zebrafish with long QT syndrome^21^,^22^,^23^,^24^. We treated MO19b injected larvae at 48 hpf with sub-phenotypic dosages (no observed effect level, NOEL, of terfenadine in wildtype larvae) of Terfenadine to sensitize the fish to developing an APD prolongation characteristic AV-block. M-mode analysis revealed that at NOEL dosages of Terfenadine (30 μM), 25% ± 4.1% of miR-19b-deficient embryos developed an AV-block, while control larvae maintained their regular heartbeat without showing any sign of AV-conduction abnormalities (Fig. 5b,c, suppl. Movie 3, 4). This strongly suggests that miR-19b deficiency leads to a prolongation of the APD below a phenotypic threshold, but significantly sensitizes hearts lacking miR-19b to developing zebrafish characteristics of a prolonged APD. To confirm the effect of miR-19b deficiency on ventricular APD, whole hearts from control and MO19b injected embryos at 48 hpf were dissected and ventricular compound APs from exogenously paced hearts were recorded (Fig. 4d). Both, APD (APD 50: 286 ms ±9.7 ms; APD 90: 349,9 ms ± 9.9 ms) and the resting membrane potential (−68.5 mV ± 2.9 mV) of control hearts were comparable to previously published data^22^. However, APD50 and APD90 in miR-19b reduced hearts were significantly prolonged by 57.4% ± 3.8% and 38.6% ± 3.5%, respectively (Fig. 4). In addition to detecting a prolonged APD, we found that most noticeably the shape of the early repolarization phase was altered. In recordings taken from control hearts, the early repolarization downward spike, or notch, before the plateau phase is visible. In contrast, recordings taken from miR-19b reduced hearts by MO19b injection show a flattened, attenuated repolarization spike, potentially indicating increased I_Na_ activity (Fig. 4d).

### miR-19b directly and indirectly regulates cardiac ion channel expression

To understand the molecular mechanism by which miR-19b modulates APD length, we analyzed the expression of cardiac ion channels known to cause APD prolongation when dysregulated or mutated in MO19b injected hearts by qPCR (Fig. 5a,b) 25. We focused particularly on channels that were upregulated upon miR-19b loss, to identify putatively directly-regulated miR-19b target transcripts. We found that the expression of several ion channels was altered, including significant upregulation of KCNE1, KCNE4, KCNJ2, SCN1B and SCN4B (Fig. 5a). Noticeably, the expression of HCN4 was not altered, suggesting normal development and function of the pacemaker cells (Fig. 5a). Interestingly, among the upregulated RNAs, transcripts harboring a predicted miR-19b binding site in their 3′-untranslated region (3′-UTR), as assessed through bioinformatics prediction, were remarkably overrepresented (Fig. 5a, marked in blue). To evaluate direct regulation, we cloned the native and full length 3′-UTRs of these putative targets into the 3′-UTR of a luciferase construct and analyzed the ability of miR-19b to repress luciferase expression in HEK293 cells. We tested the 3′-UTRs of SCN1B, SCN4B, KCNE1, KCNE4, and KCNJ2, in the respective zebrafish and human transcripts (suppl. Figure 6). miR-19b significantly repressed the expression of luciferase constructs containing the 3′-UTR of zebrafish and human SCN1B, KCNE4, and KCNJ2, and human SCN4B, but not of KCNE1 in either, human or zebrafish (Fig. 5c,d). Importantly, mutating the putative binding site in these 3′-UTRs, abolished miR-19-dependant repression of the luciferase protein.

### Reducing KCNE4 levels partially rescues bradycardia

**Figure 6:**
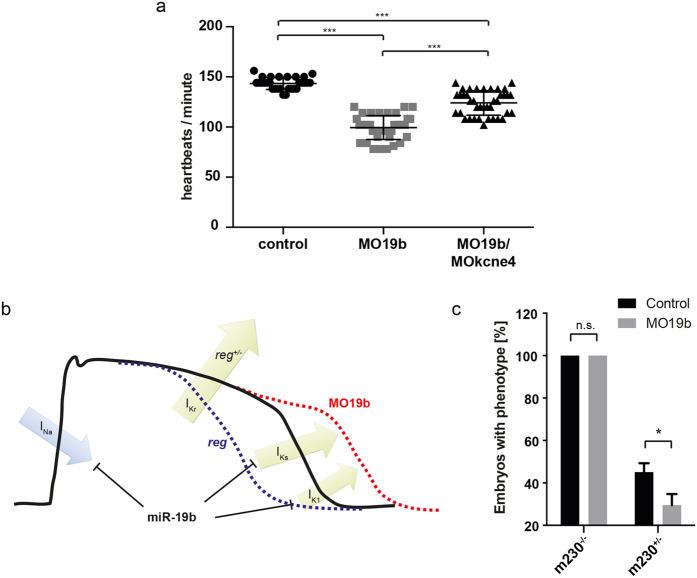
Phenotype of *reggae* mutants is partially rescued by loss of miR-19b. **(a)** Quantification of heart rate in control injected, MO19b and co-injection of MO19b and MOkcne4 (n > 50 animals from 3 independent experiments; p < 0.005 using one-way ANOVA and unpaired t-test with Welch’s correction). **(b)** Schematic illustration of miR-19b function. **(c)** Heterozygous *reggae* mutants were rescued by miR-19b knockdown (±sd; n = 73 animals from 3 independent experiments; p < 0.05).

KCNE4, together with KCNE1, are modulatory β-subunits of KCNQ1, the channel mediating the late repolarizing I_ks_-current^26^,^27^,^28^,^29^,^30^,^31^. Overexpression of KCNE4 was shown to inhibit KCNQ1 function^32^. Our results indicate a direct regulation of KCNE4 by miR-19b. If increased KCNE4 levels contribute significantly to the observed APD prolongation and the bradycardia as a result of the miR-19b reduction, we hypothesized that reducing KCNE4 levels could partly rescue miR-19b-deficiency. To test this, we co-injected sub-phenotypic amounts of a translation blocking KCNE4 MO (MOkcne4) together with MO19b, and analyzed heart rate at 48 hpf (Fig. 6a, suppl. Figure 7). While again MO19b injected larvae displayed a significantly reduced heart rate (99.46 ± 11.93 bpm) compared to control injected zebrafish (143.5 ± 6 bpm; p < 0.005), embryos co-injected with MOkcne4 showed, although still significantly lower than the control injected, a partially normalized heart rate (124 ± 12.26 bpm), indicating a partial rescue (Fig. 6a).

### miR-19b knockdown rescues short QT mutant

To further illustrate the impact of miR-19b on the electrophysiology of the zebrafish heart, we performed an additional rescue experiment. We next wanted to test whether miR-19b induced APD prolongation can be used to normalize a genetically caused pathological APD shortening. The zebrafish mutant reggae (*reg*) was previously described as a model for congenital short QT syndrome, displaying genetically caused shortening of the AP in homozygous (*reg*^−/−^) and heterozygous (*reg*^+/−^) zebrafish^33^. *reg* mutants carry a gain-of-function mutation in the zebrafish KCNH2 (zERG) potassium channel, thereby increasing I_Kr_ repolarizing currents and shortening the AP. This causes a characteristic phenotype in *reg*^−/−^ and also frequently in *reg*^+/−^ individuals, with atrial fibrillation, sinus node block, and phases with complete cessation of heart contraction^33^. We hypothesized that miR-19b loss mediated AP prolongation could be used to antagonize AP shortening in *reg* mutants and thereby to rescue the *reg* phenotype (Fig. 6b). While injection of MO19b could not rescue *reg*^−/−^ zebrafish, miR-19b loss was able to significantly prevent the development of the *reg* phenotype in almost 40% of *reg*^+/−^, often rendering mutation carriers even asymptomatic over a long period of time (Fig. 6c).

## Discussion

Here, we provide evidence that miR-19b regulates cardiac electrophysiology and we demonstrate that loss of miR-19b affects APD *in vivo*. By directly and indirectly targeting several conserved cardiac ion channels and their modulatory subunits, miR-19b functions as a fine-tuner for APD and AP shape.

The APD is determined by the balance between inward depolarizing and outward repolarizing currents during the plateau phase (Phase 2), with potassium currents playing a prominent role, including I_K1_, I_Ks_ and I_Kr_^34^. Decreased potassium currents result in impaired repolarization and thereby ultimately lead to prolonged APD. APD prolongation is a common feature in human heart failure and in animal models of cardiac hypertrophy^1^,^2^,^3^,^4^. However, the underlying molecular mechanisms causing prolongation of the APD are still incompletely understood. Some of the changes in potassium currents during heart failure are similar to congenital ion channelopathies that are known to cause LQTS. Thus, congestive heart failure can be described as a kind of acquired LQTS^35^. Furthermore, the changes in repolarizing ion channels are associated with increased risk of sudden cardiac death (SCD)^36^,^37^. miRNAs have previously been implicated to be important regulators for a number of pathological processes in the cardiovascular system, including cardiac hypertrophy, HF, and cardiac arrhythmias^5^,^6^,^38^,^39^.

miR-19b is robustly expressed in the heart and we found that miR-19b deficiency caused bradycardia frequently accompanied with reduced contractility compared to controls. Interestingly, bradycardia developed often independent of reduced systolic dysfunction, suggesting differing and uncoupled underlying mechanisms. Previous studies consistently reported bradycardia, susceptibility to arrhythmias, and cardiomyopathy in mouse and zebrafish models with dysregulated miR-19b^10^,^11^,^12^,^13^,^40^. However, the underlying mechanism largely remained unexamined. To guarantee specificity, we deployed morpholino-mediated reduction of miR-19b using several MOs and CRISPR/Cas9 gene editing to thoroughly evaluate the role of miR-19b in the heart.

miR-19b reduced hearts developed prolonged APDs. To test this hypothesis, we deployed two experimental approaches. As described previously, we used sub-phenotypic dosages of the APD-prolonging drug terfenadine and demonstrated that miR-19b-lacking hearts are sensitized to pharmacological APD prolongation and as a consequence to developing the characteristic 2^nd^ degree AV-block in zebrafish, clearly indicating prolonged APD under basal conditions^22^,^41^. Ultimately, by patch-clamp of paced whole hearts we documented significant APD prolongation. Furthermore, we found not only APD prolongation, but additionally currents during early cardiac repolarization phase were increased.

To elucidate the underlying mechanism of miR-19b-loss induced APD prolongation in an unbiased fashion, we used qPCR to assess changes in the expression of known APD prolongation-associated ion channels in hearts upon miR-19b reduction. We found several ion channel transcripts to be dysregulated, including sodium and potassium current mediating channels. Noticeably, many of the dysregulated genes were predicted targets of miR-19b. Luciferase assays, testing 3′-UTR responsiveness for miR-19b, confirmed SCN1B, SCN4B, KCNE4, and KCNJ2 as direct targets of miR-19b. Importantly, targeting of these transcripts is conserved between zebrafish and human, suggesting also conserved functional consequences of miR-19b dysregulation. In addition, KCNE1 was consistently and profoundly upregulated upon miR-19b reduction, potentially indicating indirect regulation through miR-19b.

The KCNE family is a group of proteins that function as beta regulatory subunits of KCNQ1, which is the voltage-gated potassium channel, mainly mediating I_ks_ current in cardiac tissue during repolarization. Both, KCNE1 and KCNE4, function as modulators of KCNQ1 activity^26^,^27^,^28^,^29^,^30^,^31^. Previous studies found elevated and reduced KCNE1 expression during HF^42^,^43^,^44^. Interestingly, Mansén *et al.* demonstrated that both, weaker and stronger KCNE1 expression results in reduced I_Ks_ and APD prolongation, and that the correct ratio of KCNE1 to KCNQ1 is crucial for conducting a physiological I_Ks_ current^45^. KCNE4 is another beta subunit of KCNQ1. Increased expression of KCNE4 with KCNQ1 *in vitro* resulted in complete inhibition of I_Ks_ and consequently in APD prolongation^32^. Upon miR-19b reduction we found a dramatic upregulation of both, KCNE4 and KCNE1, which might explain impaired cardiac repolarization through KCNQ1 inhibition resulting in reduced I_Ks_ and consequently in the observed prolongation of the AP and the bradycardia. Importantly, we can show that reduction of KCNE4 can partially rescue miR-19b deficiency-induced bradycardia, underlining (I) the significant contribution of KCNE4 upregulation in the development of the phenotype but also (II) that additional miR-19b targets are involved. Interestingly, Li *et al.* showed that overexpression of miR-19b in zebrafish similarly results in bradycardia^13^. Provided that both, KCNE1 and KCNE4 up- or downregulation causes KCNQ1 inhibition and APD prolongation, KCNE1/4 downregulation by miR-19b overexpression could be causing the bradycardia observed in that study^13^.

Over the last decade, evidence accumulated implying elevated late Na_v_1.5 currents, as a common feature in failing hearts^46^,^47^,^48^,^49^. The voltage-gated sodium channel predominantly regulates I_Na_ current in cardiac cells during depolarization and early repolarization phase. SCN1B, which we found directly regulated by miR-19b and upregulated upon its loss, functions as a beta subunit for Na_v_1.5^50^. It was previously shown that overexpression of SCN1B and SCN5A, the α-subunit of Na_v_1.5, increased Na_v_1.5 current amplitude and the late sodium current, I_NaL_^51^. Increased I_NaL_ ultimately leads to a prolonged APD.

Besides the described directly regulated ion channels in the heart, our qPCR data suggests additional indirectly regulated transcripts some of which are downregulated upon miR-19b reduction, including KCNA4, KCND3, SCN12B, and CACNA1C. KCNA4 and KCND3 are two channels mediating the I_to_ current^52^,^53^. Downregulation of I_to_ was previously associated with several cardiac disorders^54^,^55^,^56^,^57^,^58^,^59^. By impairment of the I_to_-current, the notch during early repolarization is decreased which might explain the elevated potential we observed during phase 1 in miR-19b deficient hearts. However, the existence of I_to_ in the zebrafish heart is still controversial^60^.

Furthermore, a previous study demonstrated that miR-19 directly targets Cx43^11^. Thus, miR-19b downregulation provokes a complex remodeling of units important for electrical activity through direct, indirect, and potentially maladaptive changes in gene expression, all contributing to the observed phenotype.

We previously described the zebrafish mutant *reggae (reg*) exhibiting features of short QT syndrome (SQTS), such as accelerated cardiac repolarization and significant APD shortening^33^. *reg* is caused by a gain-of-function mutation in the I_Kr_ conducting channel encoded by KCNH2. Importantly, kcnh2 expression is not affected by miR-19b downregulation. We subsequently tested whether miR-19b loss and consequently the resulting APD prolongation would be sufficient to prevent *reg* zebrafish from developing *reg* characteristic SQTS symptoms, including often minute-long cessation of heart contraction, atrial fibrillation, and sinus node exit block^33^. Strikingly, miR-19b reduction significantly rescued *reg*^+/−^ zebrafish, while *reg*^−/−^ remained unaffected by this treatment. Considering that miR-19b exerts a rather modulatory role in the electrical activity of the heart and loss of miR-19b results in a significant yet sub-phenotypic prolongation of the APD, the inability to rescue *reg*^−/−^ that exhibits a profound shortening of the APD with severe manifestation of a resulting phenotype appears plausible. *reg*^+/−^ develop SQTS symptoms hence display shortened APD. Here, miR-19b-loss mediated ADP prolongation was sufficient to antagonize *reg* induced APD shortening and prevented the manifestation of the characteristic phenotype in a significant proportion of *reg*^+/−^ larvae, even though *reg* induced changes in kcnh2 channel kinetics persisted. Given that the here identified direct targets of miR-19b are conserved in humans, we hypothesize that miR-19b reduction might also cause APD prolongation in human ventricles.

Taken together, our study establishes miR-19b as an important modulator of cardiac electrophysiology. Here, we provide evidence that miR-19b fine tunes the tightly balanced regulation of cardiac electrophysiology by modulating the expression of several ion-channels important for APD and AP shaping. Further we show direct regulation of corresponding human genes by miR-19b and thereby establish miR-19b as a potential candidate gene causative for human LQTS. Whether, and to which extend, miR-19b contributes to the pathophysiological electrical remodeling in the diseased myocardium should be a subject of future research.

## Methods

### Zebrafish care and maintenance

All animal experiments have been performed in accordance with the guidelines of the state of Baden-Wuerttemberg and all experimental protocols were approved by the Regierungspräsidium Karlsruhe (permit number T-90/15 and 35-9185.81/G-126/15). We used following lines: wildtype, Tg(Myl7:GFP), Tg(kdrl:GFP), Reggae-Mutants^33^. For photo and video imaging, zebrafish embryos were mounted in 2% methyl-cellulosis and analyzed under the microscope.

### Morpholino injection

Morpholinos were obtained from Gene Tools LLC. MO19 was designed to target all four miR-19 isoforms at the same time. MO19a-d were designed to target specifically one miR-19 isoform. Sequences were as follows: MO19: 5′-TCAGTTTTGCATGGATTTGCACAGC-3′; MO19a: 5′-GTAGTGCAACTATGCAAAACTAGCA-3′; MO19b: 5′-GCTGAATGCAAACCAGCAAAACTGA-3′; MO19c: 5′-GCCGGATGCAATCCTGCAAAACTCA-3′; MO19d: 5′-CTGACTGCCCACCCCGCAAAGCTGA-3′; MO363: GATTACAGATGGATACCGTGCAATT; MOkcne4: 5′-ACGACAGAAACAGGAACTGTTACCG-3′; MOcrtl: non-targeting standard control. The morpholinos were solubilized in water at a stock-concentration of 1 mM. Stock solutions were diluted with 500 mM KCl to working concentrations of 125 μM–500 μM. The injection volume was 2–5 nl/embryo, depending on the required dose. Effective doses were determined for every morpholino separately.

### CRISPR/Cas9

sgRNA was designed using chopchop (https://chopchop.rc.fas.harvard.edu/)^61^. The gene specific part of sgRNA was 5′-GCAAATCTATGCAAAACTGA-3′, excluding the NGG motif. For synthesis of sgRNA we used the pT7-gRNA Plasmid (Plasmid #46759, Addgene) and the T7-Megashortscript (AM1354, Ambion/Life Technologies). Final concentration of 150 ng/μl sgRNA and 400 ng/μl Cas9-Protein (ToolGen, Seoul, South Korea) was injected into zebrafish embryos at the single-cell-stage. T7E1-Assay (M0302S/L, New England Biolabs) was performed after PCR amplification with miR-19b specific pimers: miR-19bF: 5′-TAGGCAAACCAGCAAAACTGACC-3′ miR-19bR: 5′-AAACGGTCAGTTTTGCTGGTTTG-3′.

### Quantitive reverse transcription PCR

RNA was isolated from 10–15 embryos. Up to 1 μg of total RNA was transcribed into cDNA using miScript RT-Kit (Qiagen Cat No: 218161) or iScript (BioRad Cat No: 170–8891) for detection of miRNA or mRNA, respectively. RT-PCR was performed using miScript SYBR Green PCR Kit (Qiagen Cat No: 218073) or QuantiFast SYBR Green PCR Kit (Qiagen Cat No: 204054), according to manufacturer’s recommendations. Changes in gene expression were quantified by delta-delta-C_t_ method.

### Pharmacological treatment with Terfenadine

Terfenadine (T9652, Sigma-Aldrich) was diluted to stock concentrations of 1 mM in DMSO. 48 hpf Tg(myl7:GFP) zebrafish were treated with 10–30 μM of Terfenadin in E3-Medium. Control fish were incubated in 1% DMSO in E3-Medium. For M-Mode images fluorescing hearts were recorded over 10 seconds and single pixel lines from each frame were assembled to one image, representing the atrial or ventricular contraction over time.

### Patch-clamp

For patch-clamp recordings, only spontaneously beating and apparently healthy hearts were used. Data were recorded at room temperature using the whole cell patch-clamp technique, and action potential (AP) recordings were obtained in the current clamp configuration. Bath solution was composed of L15-Medium (Sigma-Aldrich) supplemented with 100 U/ml penicillin, 100 μg/ml streptomycin. Pipette solution consisted of 139 mM KCl, 10 mM NaCl, 0.5 mM MgCl_2_, 5 mM Mg-ATP, 0.5 mM EGTA, 0.4 mM GTPTris and 10 mM HEPES, set to pH 7.2 with KOH. Pipettes had resistances between 1.5 and 5 MΩ. Data were low-pass filtered at 1 to 2 kHz (−3 dB, four-pole Bessel filter) before digitalization at 5 to 10 kHz. Recordings were performed using a commercially available amplifier (RK-400, Bio-Logic SAS, Claix, France) and pCLAMP software (Axon Instruments) for data acquisition and analysis. Action potentials were evoked by a 5 ms sub-threshold current step. Action potential duration (APD) was measured as the duration from the overshoot to two different percentages of repolarization (50: APD50; 90: APD90).

### *In situ* hybridization of miRNAs

For detection of microRNAs in zebrafishembryos at 48 and 72 hpf we used miR-19a- and miR-19b specific miRCURY LNA detection probes (35194, 35195, Exiqon) and followed the instruction manual of the manufacturer.

### Luciferase assay

HEK293 cells in 24-well-plates were co-transfected with 1 μg of a pGL3 plasmid, containing the 3′-UTR of the indicated genes downstream of the luciferase gene, a renilla expression-plasmid, and 40 pmol of miR-19b mimic or mimic control. Two days after transfection cells were harvested and a luciferase reporter assay was performed using the Dual-Luciferase Reporter Assay System (Promega, Cat No: E1960), according to manufacturer’s recommendations. For identification of the miR-19b binding site, potential binding sites were mutated, using the QuikChange Lightning Site-Directed Mutagenesis Kit (Agilent Technologies, Cat No: 210518). Co-transfection of renilla luciferase was used to normalize firefly luciferase activity for transfection efficiency. To assay MOkcne4 efficacy we cloned the MO recognizing sequence from zebrafish kcne4 mRNA (NCBI accession number NM_001082897) 5′- and in frame to the luciferase open reading frame in the pGL3 vector (pGL3kcne4; Promega). We injected 1 nl of a solution containing 7.5 ng/μl of each, pGL3kcne4 and renilla, alone or with MOkcne4 and performed a luciferase reporter assay using the Dual-Luciferase Reporter Assay System (Promega, Cat No: E1960), according to manufacturer’s recommendations. Luciferase activity was normalized to renilla.

### Statistical analyses

Results present means of control or treated zebrafish. Most data are depicted as bar graphs or dot plots. Error bars indicate standard deviation (sd). For the determination of statistical difference between controls and treated zebrafish we used student’s t-test, except when otherwise stated. * and *** indicate p values lower than 0.05 and 0.005, respectively. Sample size is denoted as n. Statistical analysis was performed using Prism 6 (GraphPad).

## Additional Information

**How to cite this article**: Benz, A. *et al.* miR-19b Regulates Ventricular Action Potential Duration in Zebrafish. *Sci. Rep.* **6**, 36033; doi: 10.1038/srep36033 (2016).

**Publisher’s note**: Springer Nature remains neutral with regard to jurisdictional claims in published maps and institutional affiliations.

## Supplementary Material

Supplementary Information

Supplementary MOvie 1

Supplementary MOvie 2

Supplementary MOvie 3

Supplementary MOvie 4

## References

[b1] Benoist D., Stones R., Drinkhill M., Bernus O. & White E. Arrhythmogenic substrate in hearts of rats with monocrotaline-induced pulmonary hypertension and right ventricular hypertrophy. Am J Physiol Heart Circ Physiol. 300, H2230–H2237, doi: 10.1152/ajpheart.01226.2010 (2011). 21398591 PMC3119089

[b2] Helbing W. A. *et al.* ECG predictors of ventricular arrhythmias and biventricular size and wall mass in tetralogy of Fallot with pulmonary regurgitation. Heart 88, 515–519 (2002). 12381647 10.1136/heart.88.5.515 PMC1767425

[b3] Lambert V. *et al.* Right ventricular failure secondary to chronic overload in congenital heart disease: an experimental model for therapeutic innovation. J Thorac Cardiovasc Surg 139, 1197–1204, 1204 e1191, doi: 10.1016/j.jtcvs.2009.11.028 (2010). 20412956

[b4] Lee J. K. *et al.* Stage-dependent changes in membrane currents in rats with monocrotaline-induced right ventricular hypertrophy. Am J Physiol. 272, H2833–H2842 (1997). 9227563 10.1152/ajpheart.1997.272.6.H2833

[b5] Ikeda S. *et al.* Altered microRNA expression in human heart disease. Physiol Genomics 31, 367–373, doi: 10.1152/physiolgenomics.00144.2007 (2007). 17712037

[b6] van Rooij E. *et al.* A signature pattern of stress-responsive microRNAs that can evoke cardiac hypertrophy and heart failure. Proc Natl Acad Sci USA 103, 18255–18260, doi: 10.1073/pnas.0608791103 (2006). 17108080 PMC1838739

[b7] Mogilyansky E. & Rigoutsos I. The miR-17/92 cluster: a comprehensive update on its genomics, genetics, functions and increasingly important and numerous roles in health and disease. Cell Death Differ. 20, 1603–1614, doi: 10.1038/cdd.2013.125 (2013). 24212931 PMC3824591

[b8] Ota A. *et al.* Identification and characterization of a novel gene, C13orf25, as a target for 13q31-q32 amplification in malignant lymphoma. Cancer Res. 64, 3087–3095 (2004). 15126345 10.1158/0008-5472.can-03-3773

[b9] Ventura A. *et al.* Targeted deletion reveals essential and overlapping functions of the miR-17 through 92 family of miRNA clusters. Cell 132, 875–886, doi: 10.1016/j.cell.2008.02.019 (2008). 18329372 PMC2323338

[b10] van Almen G. C. *et al.* MicroRNA-18 and microRNA-19 regulate CTGF and TSP-1 expression in age-related heart failure. Aging Cell 10, 769–779, doi: 10.1111/j.1474-9726.2011.00714.x (2011). 21501375 PMC3193380

[b11] Danielson L. S. *et al.* Cardiovascular dysregulation of miR-17-92 causes a lethal hypertrophic cardiomyopathy and arrhythmogenesis. FASEB J 27, 1460–1467, doi: 10.1096/fj.12-221994 (2013). 23271053 PMC3606524

[b12] Song D. W., Ryu J. Y., Kim J. O., Kwon E. J. & Kim do H. The miR-19a/b family positively regulates cardiomyocyte hypertrophy by targeting atrogin-1 and MuRF-1. Biochem J 457, 151–162, doi: 10.1042/BJ20130833 (2014). 24117217

[b13] Li M. *et al.* Overexpression of miR-19b impairs cardiac development in zebrafish by targeting ctnnb1. Cell Physiol Biochem. 33, 1988–2002, doi: 10.1159/000362975 (2014). 25034767

[b14] Verkerk A. O. & Remme C. A. Zebrafish: a novel research tool for cardiac (patho)electrophysiology and ion channel disorders. Front Physiol. 3, 255, doi: 10.3389/fphys.2012.00255 (2012). 22934012 PMC3429032

[b15] Kloosterman W. P., Lagendijk A. K., Ketting R. F., Moulton J. D. & Plasterk R. H. Targeted inhibition of miRNA maturation with morpholinos reveals a role for miR-375 in pancreatic islet development. PLoS Biol 5, e203, doi: 10.1371/journal.pbio.0050203 (2007). 17676975 PMC1925136

[b16] Hassel D. *et al.* MicroRNA-10 regulates the angiogenic behavior of zebrafish and human endothelial cells by promoting vascular endothelial growth factor signaling. Circ Res. 111, 1421–1433, doi: 10.1161/CIRCRESAHA.112.279711 (2012). 22955733 PMC3525481

[b17] Chiavacci E. *et al.* MicroRNA 19a replacement partially rescues fin and cardiac defects in zebrafish model of Holt Oram syndrome. Sci Rep. 5, 18240, doi: 10.1038/srep18240 (2015). 26657204 PMC4677400

[b18] Yin R., Bao W., Xing Y., Xi T. & Gou S. MiR-19b-1 inhibits angiogenesis by blocking cell cycle progression of endothelial cells. Biochem Biophys Res Commun. 417, 771–776, doi: 10.1016/j.bbrc.2011.12.032 (2012). 22197821

[b19] Landskroner-Eiger S. *et al.* Endothelial miR-17 approximately 92 cluster negatively regulates arteriogenesis via miRNA-19 repression of WNT signaling. Proc Natl Acad Sci USA 112, 12812–12817, doi: 10.1073/pnas.1507094112 (2015). 26417068 PMC4611600

[b20] Tang Y. *et al.* The role of miR-19b in the inhibition of endothelial cell apoptosis and its relationship with coronary artery disease. Sci Rep. 5, 15132, doi: 10.1038/srep15132 (2015). 26459935 PMC4602285

[b21] Milan D. J., Peterson T. A., Ruskin J. N., Peterson R. T. & MacRae C. A. Drugs that induce repolarization abnormalities cause bradycardia in zebrafish. Circulation 107, 1355–1358 (2003). 12642353 10.1161/01.cir.0000061912.88753.87

[b22] Arnaout R. *et al.* Zebrafish model for human long QT syndrome. Proc Natl Acad Sci USA 104, 11316–11321, doi: 10.1073/pnas.0702724104 (2007). 17592134 PMC2040896

[b23] Langheinrich U., Vacun G. & Wagner T. Zebrafish embryos express an orthologue of HERG and are sensitive toward a range of QT-prolonging drugs inducing severe arrhythmia. Toxicol Appl Pharmacol. 193, 370–382 (2003). 14678746 10.1016/j.taap.2003.07.012

[b24] Leong I. U., Skinner J. R., Shelling A. N. & Love D. R. Zebrafish as a model for long QT syndrome: the evidence and the means of manipulating zebrafish gene expression. Acta Physiol (Oxf) 199, 257–276, doi: 10.1111/j.1748-1716.2010.02111.x (2010). 20331541

[b25] Schwartz P. J. Introduction to the arrhythmogenic disorders of genetic origin series. Circ Arrhythm Electrophysiol. 5, 604–605, doi: 10.1161/CIRCEP.112.971846 (2012). 22715239

[b26] Tinel N., Diochot S., Borsotto M., Lazdunski M. & Barhanin J. KCNE2 confers background current characteristics to the cardiac KCNQ1 potassium channel. EMBO J 19, 6326–6330, doi: 10.1093/emboj/19.23.6326 (2000). 11101505 PMC305874

[b27] Schroeder B. C. *et al.* A constitutively open potassium channel formed by KCNQ1 and KCNE3. Nature 403, 196–199, doi: 10.1038/35003200 (2000). 10646604

[b28] Neyroud N. *et al.* A novel mutation in the potassium channel gene KVLQT1 causes the Jervell and Lange-Nielsen cardioauditory syndrome. Nat Genet 15, 186–189, doi: 10.1038/ng0297-186 (1997). 9020846

[b29] Sanguinetti M. C. *et al.* Coassembly of K(V)LQT1 and minK (IsK) proteins to form cardiac I(Ks) potassium channel. Nature 384, 80–83, doi: 10.1038/384080a0 (1996). 8900283

[b30] Barhanin J. *et al.* K(V)LQT1 and lsK (minK) proteins associate to form the I(Ks) cardiac potassium current. Nature 384, 78–80, doi: 10.1038/384078a0 (1996). 8900282

[b31] Sanguinetti M. C. & Jurkiewicz N. K. Two components of cardiac delayed rectifier K+ current. Differential sensitivity to block by class III antiarrhythmic agents. J Gen Physiol 96, 195–215 (1990). 2170562 10.1085/jgp.96.1.195 PMC2228985

[b32] Grunnet M. *et al.* KCNE4 is an inhibitory subunit to the KCNQ1 channel. J Physiol. 542, 119–130 (2002). 12096056 10.1113/jphysiol.2002.017301 PMC2290389

[b33] Hassel D. *et al.* Deficient zebrafish ether-a-go-go-related gene channel gating causes short-QT syndrome in zebrafish reggae mutants. Circulation 117, 866–875, doi: 10.1161/CIRCULATIONAHA.107.752220 (2008). 18250272

[b34] Carmeliet E. K+ channels in cardiac cells: mechanisms of activation, inactivation, rectification and K+e sensitivity. Pflugers Arch 414 Suppl 1, S88–S92 (1989). 2674895 10.1007/BF00582254

[b35] Choy A. M. *et al.* Normalization of acquired QT prolongation in humans by intravenous potassium. Circulation 96, 2149–2154 (1997). 9337183 10.1161/01.cir.96.7.2149

[b36] Tomaselli G. F. *et al.* Sudden cardiac death in heart failure. The role of abnormal repolarization. Circulation 90, 2534–2539 (1994). 7955213 10.1161/01.cir.90.5.2534

[b37] Day C. P., McComb J. M. & Campbell R. W. QT dispersion: an indication of arrhythmia risk in patients with long QT intervals. Br Heart J 63, 342–344 (1990). 2375895 10.1136/hrt.63.6.342 PMC1024518

[b38] Lu Y. *et al.* MicroRNA-328 contributes to adverse electrical remodeling in atrial fibrillation. Circulation 122, 2378–2387, doi: 10.1161/CIRCULATIONAHA.110.958967 (2010). 21098446

[b39] Zhao Y. *et al.* Dysregulation of cardiogenesis, cardiac conduction, and cell cycle in mice lacking miRNA-1-2. Cell 129, 303–317, doi: 10.1016/j.cell.2007.03.030 (2007). 17397913

[b40] Chen J. *et al.* mir-17-92 cluster is required for and sufficient to induce cardiomyocyte proliferation in postnatal and adult hearts. Circ Res 112, 1557–1566, doi: 10.1161/CIRCRESAHA.112.300658 (2013). 23575307 PMC3756475

[b41] Kopp R., Schwerte T. & Pelster B. Cardiac performance in the zebrafish breakdance mutant. J Exp Biol 208, 2123–2134, doi: 10.1242/jeb.01620 (2005). 15914656

[b42] Tsuji Y., Zicha S., Qi X. Y., Kodama I. & Nattel S. Potassium channel subunit remodeling in rabbits exposed to long-term bradycardia or tachycardia: discrete arrhythmogenic consequences related to differential delayed-rectifier changes. Circulation 113, 345–355, doi: 10.1161/CIRCULATIONAHA.105.552968 (2006). 16432066

[b43] Li X. *et al.* Bisoprolol reverses down-regulation of potassium channel proteins in ventricular tissues of rabbits with heart failure. J Biomed Res. 25, 274–279, doi: 10.1016/S1674-8301(11)60037-7 (2011). 23554701 PMC3597065

[b44] Watanabe E. *et al.* Upregulation of KCNE1 induces QT interval prolongation in patients with chronic heart failure. Circ J 71, 471–478 (2007). 17384445 10.1253/circj.71.471

[b45] Mansen A. *et al.* Thyroid hormone receptor alpha can control action potential duration in mouse ventricular myocytes through the KCNE1 ion channel subunit. Acta Physiol (Oxf) 198, 133–142, doi: 10.1111/j.1748-1716.2009.02052.x (2010). 19832729

[b46] Maltsev V. A., Silverman N., Sabbah H. N. & Undrovinas A. I. Chronic heart failure slows late sodium current in human and canine ventricular myocytes: implications for repolarization variability. Eur J Heart Fail 9, 219–227, doi: 10.1016/j.ejheart.2006.08.007 (2007). 17067855 PMC1847560

[b47] Maltsev V. A. & Undrovinas A. I. A multi-modal composition of the late Na+ current in human ventricular cardiomyocytes. Cardiovasc Res. 69, 116–127, doi: 10.1016/j.cardiores.2005.08.015 (2006). 16223473 PMC1435371

[b48] Undrovinas A. I., Maltsev V. A. & Sabbah H. N. Repolarization abnormalities in cardiomyocytes of dogs with chronic heart failure: role of sustained inward current. Cell Mol Life Sci. 55, 494–505 (1999). 10228563 10.1007/s000180050306 PMC11147047

[b49] Valdivia C. R. *et al.* Increased late sodium current in myocytes from a canine heart failure model and from failing human heart. J Mol Cell Cardiol. 38, 475–483, doi: 10.1016/j.yjmcc.2004.12.012 (2005). 15733907

[b50] Liu M., Yang K. C. & Dudley S. C. Jr. Cardiac sodium channel mutations: why so many phenotypes? Nat Rev Cardiol. 11, 607–615, doi: 10.1038/nrcardio.2014.85 (2014). 24958080 PMC4878851

[b51] Maltsev V. A., Kyle J. W. & Undrovinas A. Late Na+ current produced by human cardiac Na+ channel isoform Nav1.5 is modulated by its beta1 subunit. J Physiol Sci. 59, 217–225, doi: 10.1007/s12576-009-0029-7 (2009). 19340536 PMC2744134

[b52] Dixon J. E. *et al.* Role of the Kv4.3 K+ channel in ventricular muscle. A molecular correlate for the transient outward current. Circ Res. 79, 659–668 (1996). 8831489 10.1161/01.res.79.4.659

[b53] Kassiri Z., Hajjar R. & Backx P. H. Molecular components of transient outward potassium current in cultured neonatal rat ventricular myocytes. J Mol Med (Berl) 80, 351–358, doi: 10.1007/s00109-002-0325-7 (2002). 12072910

[b54] Brundel B. J. *et al.* Alterations in potassium channel gene expression in atria of patients with persistent and paroxysmal atrial fibrillation: differential regulation of protein and mRNA levels for K+ channels. J Am Coll Cardiol. 37, 926–932 (2001). 11693772 10.1016/s0735-1097(00)01195-5

[b55] Gaborit N. *et al.* Human atrial ion channel and transporter subunit gene-expression remodeling associated with valvular heart disease and atrial fibrillation. Circulation 112, 471–481, doi: 10.1161/CIRCULATIONAHA.104.506857 (2005). 16027256

[b56] Grammer J. B., Bosch R. F., Kuhlkamp V. & Seipel L. Molecular remodeling of Kv4.3 potassium channels in human atrial fibrillation. J Cardiovasc Electrophysiol. 11, 626–633 (2000). 10868735 10.1111/j.1540-8167.2000.tb00024.x

[b57] Ji Q. *et al.* Expression changes of ionic channels in early phase of cultured rat atrial myocytes induced by rapid pacing. J Cardiothorac Surg. 8, 194, doi: 10.1186/1749-8090-8-194 (2013). 24074263 PMC3851479

[b58] Kaab S. *et al.* Molecular basis of transient outward potassium current downregulation in human heart failure: a decrease in Kv4.3 mRNA correlates with a reduction in current density. Circulation 98, 1383–1393 (1998). 9760292 10.1161/01.cir.98.14.1383

[b59] Rossow C. F., Minami E., Chase E. G., Murry C. E. & Santana L. F. NFATc3-induced reductions in voltage-gated K+ currents after myocardial infarction. Circ Res. 94, 1340–1350, doi: 10.1161/01.RES.0000128406.08418.34 (2004). 15087419

[b60] Nemtsas P., Wettwer E., Christ T., Weidinger G. & Ravens U. Adult zebrafish heart as a model for human heart? An electrophysiological study. J Mol Cell Cardiol. 48, 161–171, doi: 10.1016/j.yjmcc.2009.08.034 (2010). 19747484

[b61] Montague T. G., Cruz J. M., Gagnon J. A., Church G. M. & Valen E. CHOPCHOP: a CRISPR/Cas9 and TALEN web tool for genome editing. Nucleic Acids Res. 42, W401–W407, doi: 10.1093/nar/gku410 (2014). 24861617 PMC4086086

