# Supplementary material for: miR-19b Regulates Ventricular Action Potential Duration in Zebrafish
Source: Sci Rep. 2016 Nov 2;6:36033. doi: 10.1038/srep36033 (PMC5090966; doi:10.1038/srep36033)
Supplement: Supplementary Information [file srep36033-s1.pdf]

Supplementary Information

miR-19b Regulates Ventricular Action Potential Duration in Zebrafish

Alexander Benz, Mandy Kossack, Dominik Auth, Claudia Seyler, Edgar Zitron, Lonny Juergensen, Hugo A. Katus and David Hassel

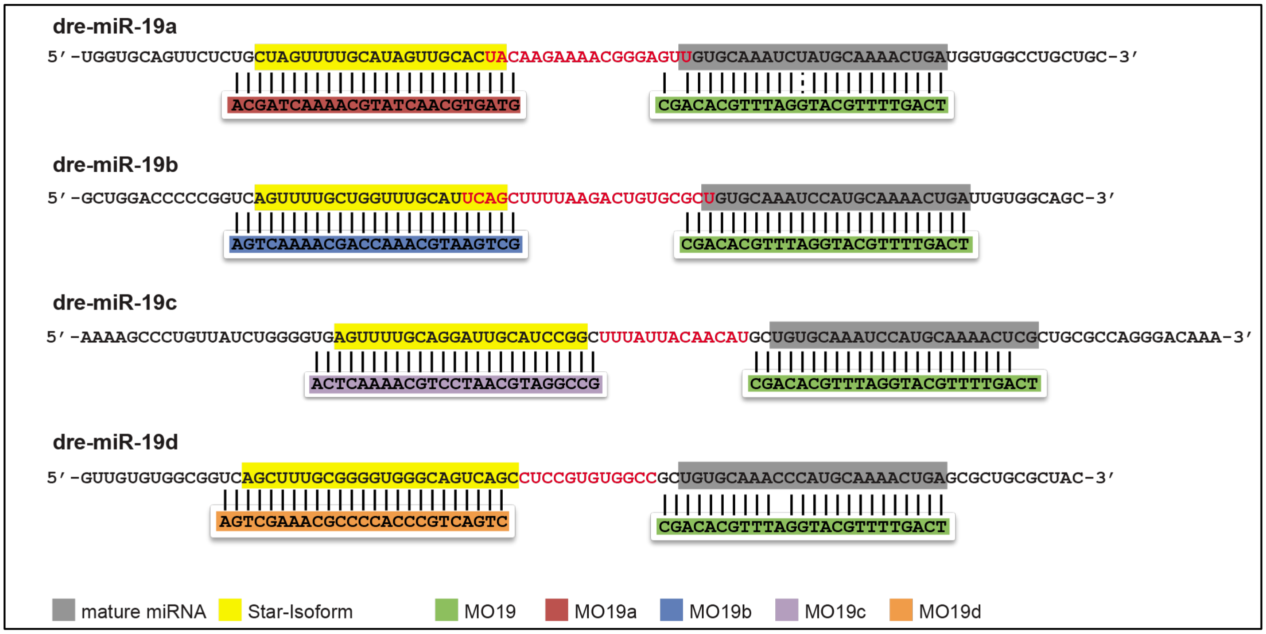

Supplementary Figure 1: Design of morpholinos for knockdown of miR-19-Isoforms. Based on the conserved homology of miR-19-Isoforms, MO19 (green) was designed to target and reduce expression of all four miR-19-Isoforms. For the specific knockdown of single miR-19-Isoforms, morpholinos were designed to target the less conserved Star-Isoform region (MO19a - red, MO19b - blue, MO19c – violet and MO19d - orange). The Sequence of the mature Sequence and the Star-isoform are highlighted in grey and in yellow, respectively.

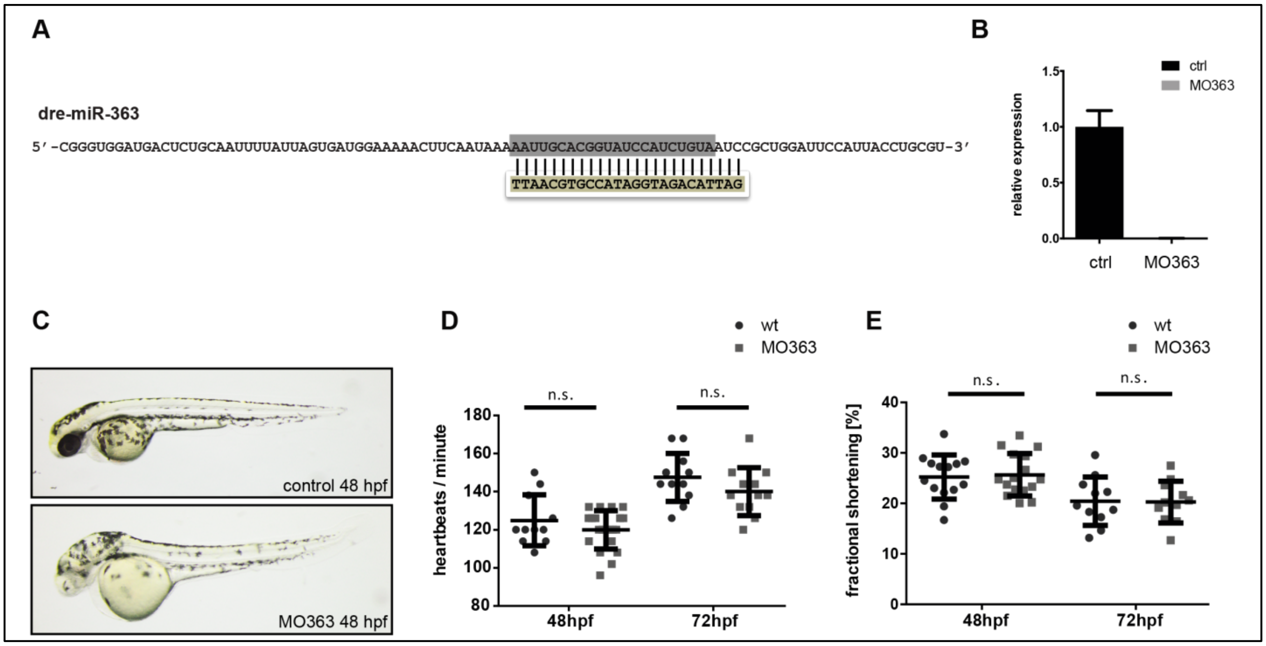

Supplementary Figure 2: Reduction of miR-363 (A) The morpholino targeting miR-363 (MO363 - green) was designed to bind the mature sequence of miR-363. **(B)** The Expression of miR-363 was reduced by 99 % by MO363-deficiency in zebrafish at 48 hpf (± sd; n=45 animals from 3 independent experiments; p<0,005). **(C)** miR-363 deficiency resulted in impaired eye development with reduced eye size up to complete loss of eyes. **(D,E)** Loss of miR-363 did not alter heart rate or contractility (± sd; n≥11).

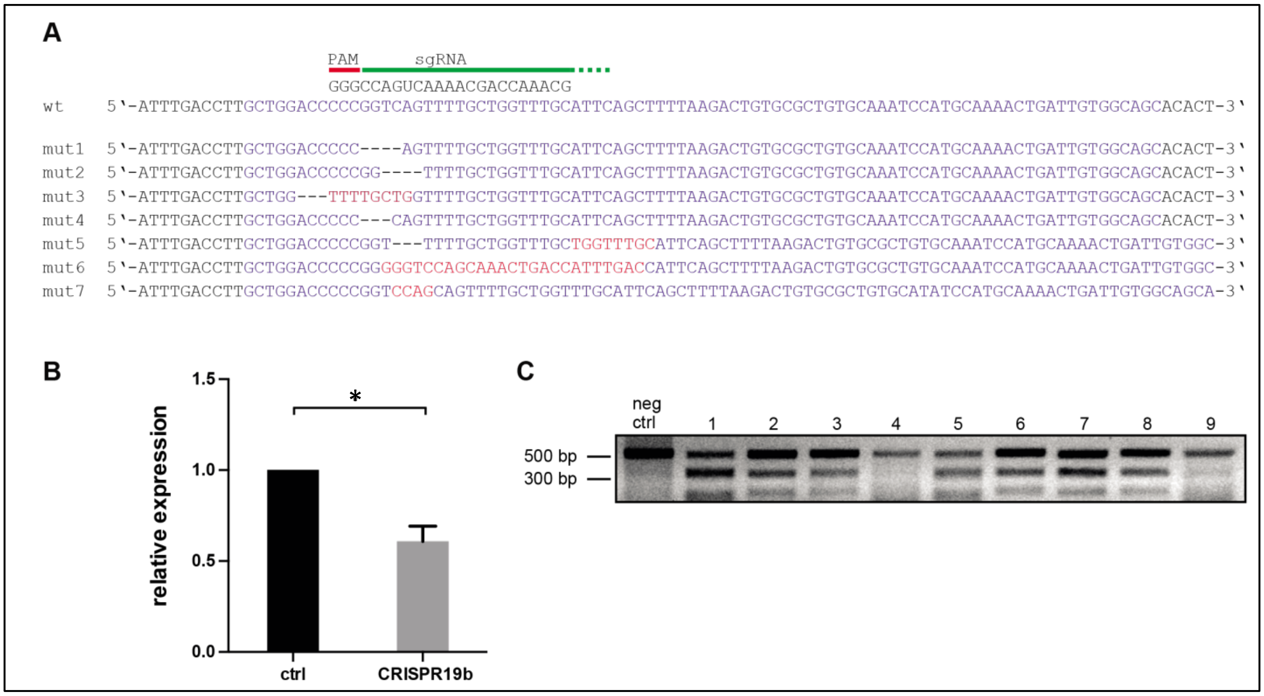

Supplementary Figure 3: Reduction of miR-19b by CRISPR/Cas9 (A) sgRNA (green) was designed to target the drosha-site of miR-19b to impair miR-19b maturation. Examples of resulting mutations are listed as mut1-mut7. Importantly, genomic sequence of miR-19a locus is unaffected. **(B)** Expression analysis of miR-19b by qRT-PCR in Crispants at 48 hpf revealed significant downregulation of miR-19b to an expected level of 50-60 % similar to morpholino mediated knockdown due to the high sequence similarity and the inability of the qPCR assay used to distinguish both isoforms (± sd; n=3 from 15 pooled embryos per sample; p<0,05). **(C)** Mutation efficiency of the CRISPR/Cas9 System was assessed by T7E1 Assay. Out of 9 Embryos 7 (embryos 1-3 and 5-8) showed to be positive for a mutation in the miR-19b gene. Neg ctrl; uninjected control embryo after T7E1 assay.

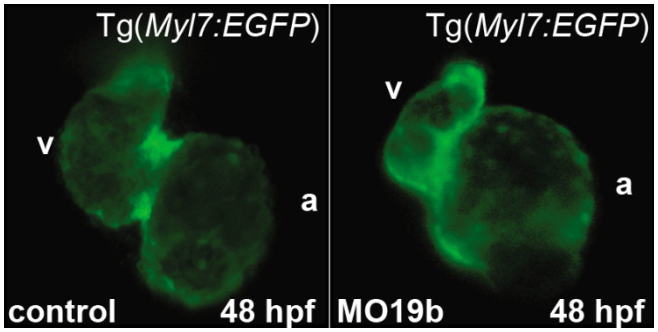

Supplementary Figure 4: Heart Morphology after MO19b Application The overall morphology of the hearts in *Tg(myl7:EGFP)* zebrafish appeared to be not affected by reduction of miR-19b. v=ventricle; a=atrium.

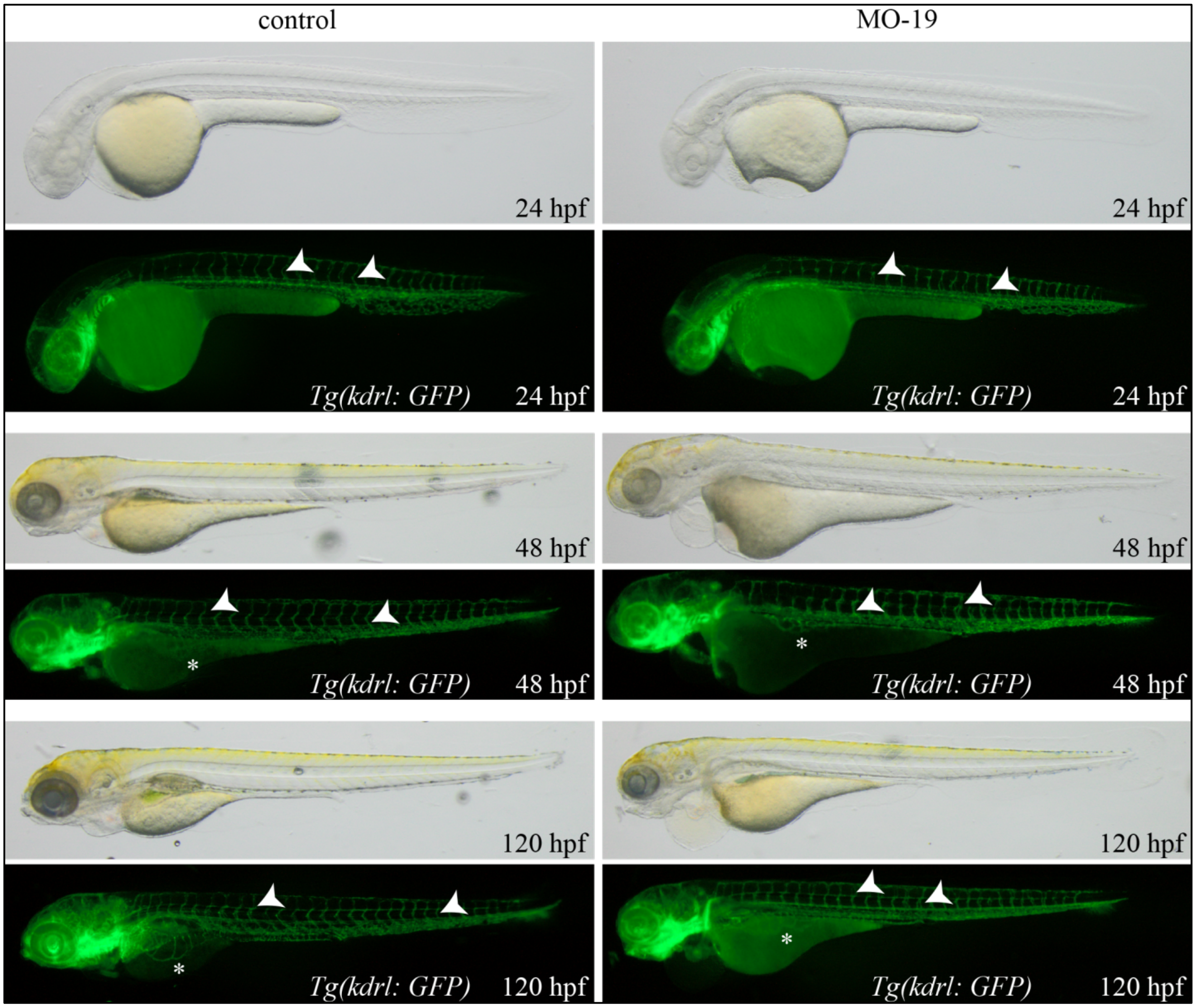

Supplementary Figure 5: miR-19b-deficient zebrafish do not display defects in angiogenesis. Lateral brightfield (upper) and fluorescent (lower) images of control (left column) and MO-19 injected (right column) *Tg(kdrl:GFP)* transgenic zebrafish labeling all vascular endothelial cells in green at indicated timepoints. The fish display typical characteristics of heart dysfunction with blood congestion at the inflow tract and cardiac edema. However, the development of intersegmental vessels (arrowheads) and sub intestinal vessels (stars) was not noticeably impaired during the first five days of development.

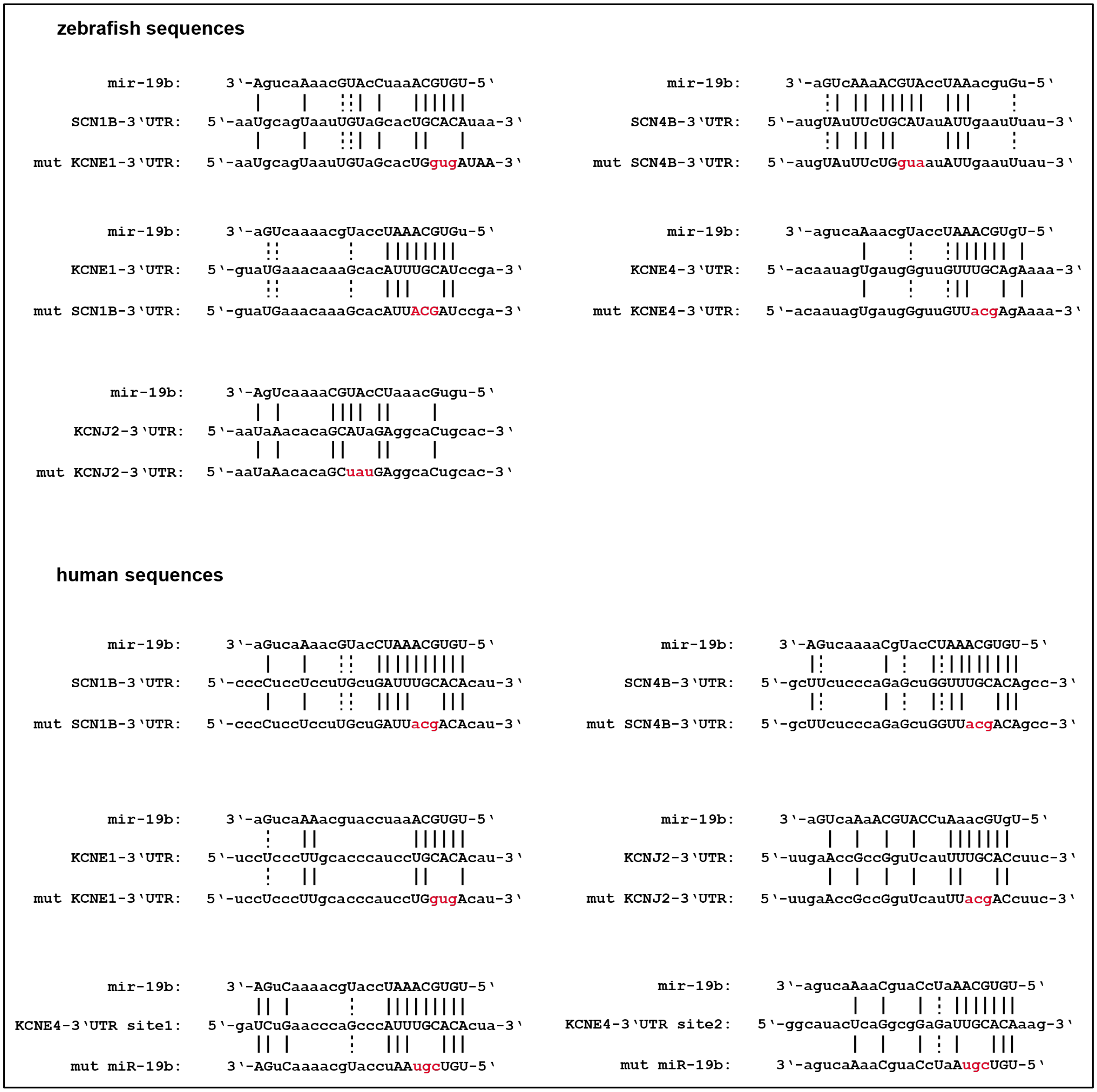

Supplementary Figure 6: Binding site for miR-19b in 3'-UTRs of potential targets. The 3'-UTR of all putative **(A)** zebrafish and **(B)** human miR-19b target genes were used for reporter gene luciferase assays. The predicted binding sites for miR-19b for each 3'-UTR are indicated. As a control, the binding site was mutated (red letters) to impair miR-19b binding and to prevent miR-19b-induced repression.

Supplementary Figure 7

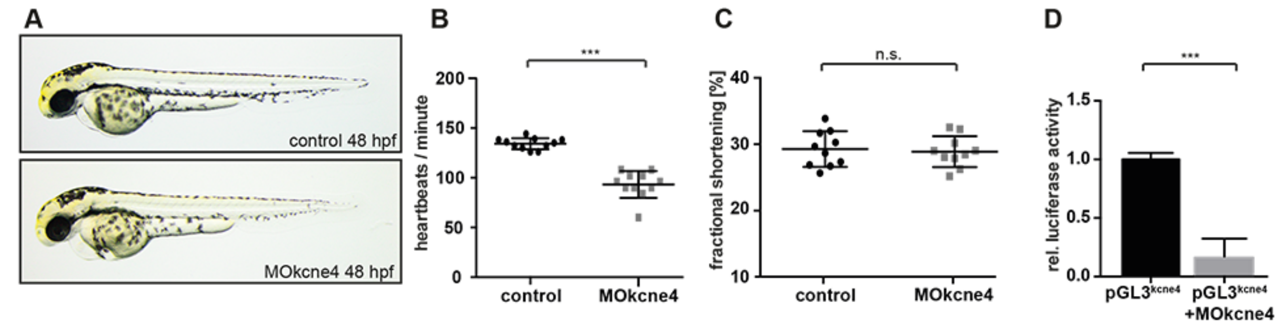

Supplementary Figure 7: MOkcne4 testing and validation. (A) Lateral view of control- and MOkcne4 injected zebrafish embryos at 48 hpf. Note the pericardial edema and blood congestion at the inflow tract. **(B, C)** Quantification of heart rate **(B)** in control- and MOkcne4-injected embryos at 48 hpf documents a significant reduction in heart rate by KCNE4 knockdown without affecting contractility **(C)**. **(D)** Luciferase reporter assay with the MOkcne4 recognizing translational start sequence from zebrafish kcne4 mRNA 5'- and in frame to the Luciferase open reading frame of the luciferase gene ($pGL3^{kcne4}$). Coinjection of this construct with MOkcne4 ($pGL3^{kcne4}$+MOkcne4) significantly repressed luciferase activity. Luciferase expression was normalized to renilla expression. (± sd; ***, p<0.005; three independent experiments; unpaired t-test with Welch's correction).

Supplementary Movie 1: Control injected larvae at 48 hpf. Head to the upper right. Tail to the lower left. Note the regular heart beat.

Supplementary Movie 2: MO19b-injected larvae at 48 hpf. Head to the top. Note the pericardial edema and the slow heart beat.

Supplementary Movie 3: Control injected larvae at 48 hpf treated with 30 µM terfenadine. Head to the upper right. Tail to the lower left.

Supplementary Movie 4: MO19b-injected larvae at 48 hpf treated with 30 µM Terfenadine. Head to the upper right. Tail to the lower left. Note the 2:1 AV-Block.
